# The Wnt Serpentine Receptor Frizzled-9 Regulates New Bone Formation in Fracture Healing

**DOI:** 10.1371/journal.pone.0084232

**Published:** 2013-12-31

**Authors:** Aline Heilmann, Thorsten Schinke, Ronny Bindl, Tim Wehner, Anna Rapp, Melanie Haffner-Luntzer, Claudia Nemitz, Astrid Liedert, Michael Amling, Anita Ignatius

**Affiliations:** 1 Institute of Orthopaedic Research and Biomechanics, Center of Musculoskeletal Research, University of Ulm, Ulm, Germany; 2 Department of Osteology and Biomechanics, University Medical Center Hamburg-Eppendorf, Hamburg, Germany; Inserm U606 and University Paris Diderot, France

## Abstract

Wnt signaling is a key regulator of bone metabolism and fracture healing. The canonical Wnt/β-catenin pathway is regarded as the dominant mechanism, and targeting this pathway has emerged as a promising strategy for the treatment of osteoporosis and poorly healing fractures. In contrast, little is known about the role of non-canonical Wnt signaling in bone. Recently, it was demonstrated that the serpentine receptor Fzd9, a Wnt receptor of the Frizzled family, is essential for osteoblast function and positively regulates bone remodeling via the non-canonical Wnt pathway without involving β-catenin-dependent signaling. Here we investigated whether the Fzd9 receptor is essential for fracture healing using a femur osteotomy model in *Fzd9* ^−/−^ mice. After 10, 24 and 32 days the fracture calli were analyzed using biomechanical testing, histomorphometry, immunohistochemistry, and micro-computed tomography. Our results demonstrated significantly reduced amounts of newly formed bone at all investigated healing time points in the absence of *Fzd9* and, accordingly, a decreased mechanical competence of the callus tissue in the late phase of fracture healing. In contrast, cartilage formation and numbers of osteoclasts degrading mineralized matrix were unaltered. β-Catenin immunolocalization showed that canonical Wnt-signaling was not affected in the absence of *Fzd9* in osteoblasts as well as in proliferating and mature chondrocytes within the fracture callus. The expression of established differentiation markers was not altered in the absence of *Fzd9*, whereas chemokines Ccl2 and Cxcl5 seemed to be reduced. Collectively, our results suggest that non-canonical signaling via the Fzd9 receptor positively regulates intramembranous and endochondral bone formation during fracture healing, whereas it does not participate in the formation of cartilage or in the osteoclastic degradation of mineralized matrix. The finding that Fzd9, in addition to its role in physiological bone remodeling, regulates bone repair may have implications for the development of treatments for poorly or non-healing fractures.

## Introduction

Bone is continuously remodeled through the finely tuned interaction between bone forming osteoblasts and bone resorbing osteoclasts [1]. An imbalance between bone formation and resorption can induce skeletal disorders, including osteoporosis, a common disease characterized by a systemic deterioration of bone mass and structure, and by fragility fractures, which are often associated with complications, such as impaired healing [2]. Due to the high socioeconomic burden of this disease, research aims to better understand the molecular mechanisms regulating bone cell activities and to identify novel therapeutic targets for the treatment of osteoporosis and osteoporotic fractures.

In recent years, the function of Wnt-dependent signal transduction has attracted considerable interest in bone biology. Commonly, the complex Wnt-signaling pathways are subdivided into the canonical and non-canonical pathways [3], [4], [5]. Signaling through the canonical pathway is initiated by the binding of Wnt ligands to Frizzled (Fzd) receptors and low-density lipoprotein receptor-related protein-5/6 (Lrp5/6) co-receptors. Downstream signaling involves the stabilization of β-catenin and its translocation to the nucleus, where it forms a transcriptional complex with T-cell factor (TCF)/lymphoid enhancer-binding factor (LEF) to regulate the transcription of Wnt target genes. Non-canonical signaling does not involve Lrp5/6 and is β-catenin independent [3], [5]. The canonical Wnt/β-catenin pathways in particular are regarded to be of crucial importance in bone biology. This became evident from specific human bone pathologies, which were associated with aberrant Wnt/β-catenin signaling. Inactivating mutations of LRP5 induce the osteoporosis-pseudoglioma syndrome in humans [6], whereas gain-of-function mutations of LRP5 result in a high bone mass phenotype because of increased osteoblast activity [7], [8]. Polymorphisms in the LRP5 gene have been reported to be associated with decreased bone mineral density (BMD) and osteoporotic fractures [9]. Furthermore, loss of SOST, a negative regulator of osteoblast activity produced by osteocytes, which binds to LRP5 and inhibits Wnt signaling [10], leads to high bone mass disorders, such as van Buchem disease or sclerostosis [10], [11].These observations, together with a large number of studies on mouse models, which demonstrate that altered Wnt signal transduction displays bone remodeling phenotypes, suggest that Wnt-signaling pathways are of crucial importance for the regulation of osteoblast and osteoclast activity [3], [5].

It is therefore not surprising that Wnt signaling also has an important function in fracture repair. Fracture healing occurs in the closely linked phases of inflammation, repair and remodeling. The repair phase involves the recruitment of precursor cells, their proliferation and differentiation, and bone formation by intramembranous and endochondral ossification. In the final phase of bone healing, the initial woven bone is remodeled by the interaction of osteoclasts and osteoblasts [12], [13]. It was demonstrated that several Wnt ligands, including Wnt4, Wnt5a, Wnt10b, Wnt11 and Wnt13, Wnt receptors, including Fzd1, 2, 4 and 5, the co-receptors Lrp5 and Lrp6, β-catenin and Wnt target genes, including *Runx2*, a transcription factor associated with osteoblast differentiation, were up-regulated in the facture callus during bone regeneration [14], [15], [16], [17], [18]. Activation of Wnt signaling by the administration of the canonical Wnt agonist Wnt3a [19], LiCl, an inhibitor of glycogen synthase kinase 3β (GSK3β) phosphorylation of β-catenin for proteosomal degradation [15], or neutralizing antibodies against the canonical Wnt inhibitors Dkk1 [20] and SOST [21], [22], [23] were shown to improve bone healing in mice. In contrast, the inhibition of Wnt signaling by treatment with recombinant Dkk1 [15], adenoviral overexpression of Dkk1 [24] or the deletion of the Wnt co-receptor Lrp5 [25] impaired fracture healing. These studies implicate that canonical Wnt/β-catenin signaling might be the dominant mechanism in bone repair. However, the up-regulation of the non-canonical Wnt agonists Wnt5a and Wnt11 during fracture healing suggest that non-canonical pathways may also be important, however, their function in bone repair has not yet been investigated [4].

Due to their tremendous importance in bone formation, targeting of Wnt-signaling pathways has emerged as a promising strategy for the treatment of bone disorders, such as osteoporosis and poorly or non-healing fractures. Neutralizing antibodies against the Wnt inhibitors Dkk1 and SOST exhibited promising results in preclinical and clinical studies [26]. To broaden the spectrum of effective drugs in clinical use, further research regarding possible new Wnt-signaling targets is needed. In this regard, the transmembrane Fzd receptors are of particular interest, because they belong to the serpentine receptor family, representing the major class of target proteins for currently available drugs [27]. Recently, we provided the first evidence for a physiological role of *Fzd9*, one of the ten known *Fzd* genes, as a positive regulator of bone formation [28]. We found that *Fzd9* is the only *Fzd* gene to be significantly up-regulated during the early stages of osteoblast differentiation, and that *Fzd9*-deficient mice displayed a cell-autonomous osteoblast defect of low bone formation. We further demonstrated that *Fzd9* regulates osteoblast function via non-canonical Wnt-signaling pathways [28]. Notably, there is evidence that FZD9 may also be involved in the regulation of bone formation in humans. FZD9 is one of the genes, whose homozygous deletion in humans induces Williams-Beuren syndrome, a disorder associated with multiple manifestations, including low bone mass [29], [30], [31].

In the present study, we investigated whether signaling through the Fzd9 receptor regulates new bone formation in fracture healing in mice. We found that new bone formation both in the early and later stages of fracture healing was significantly impaired in the absence of Fzd9, suggesting that non-canonical Wnt signaling via Fzd9 might positively regulate bone repair, a finding that may have implications for the treatment of delayed fracture healing.

## Results

### *Fzd9* Deficiency Impaired New Bone Formation in the Fracture Callus

The formation of new tissue in the fracture callus was evaluated in the early healing period at day 10 by histomorphometry, and in the later stages of healing (at days 24 and 32) using histomorphometry and µCT analysis.

**Figure 1:**
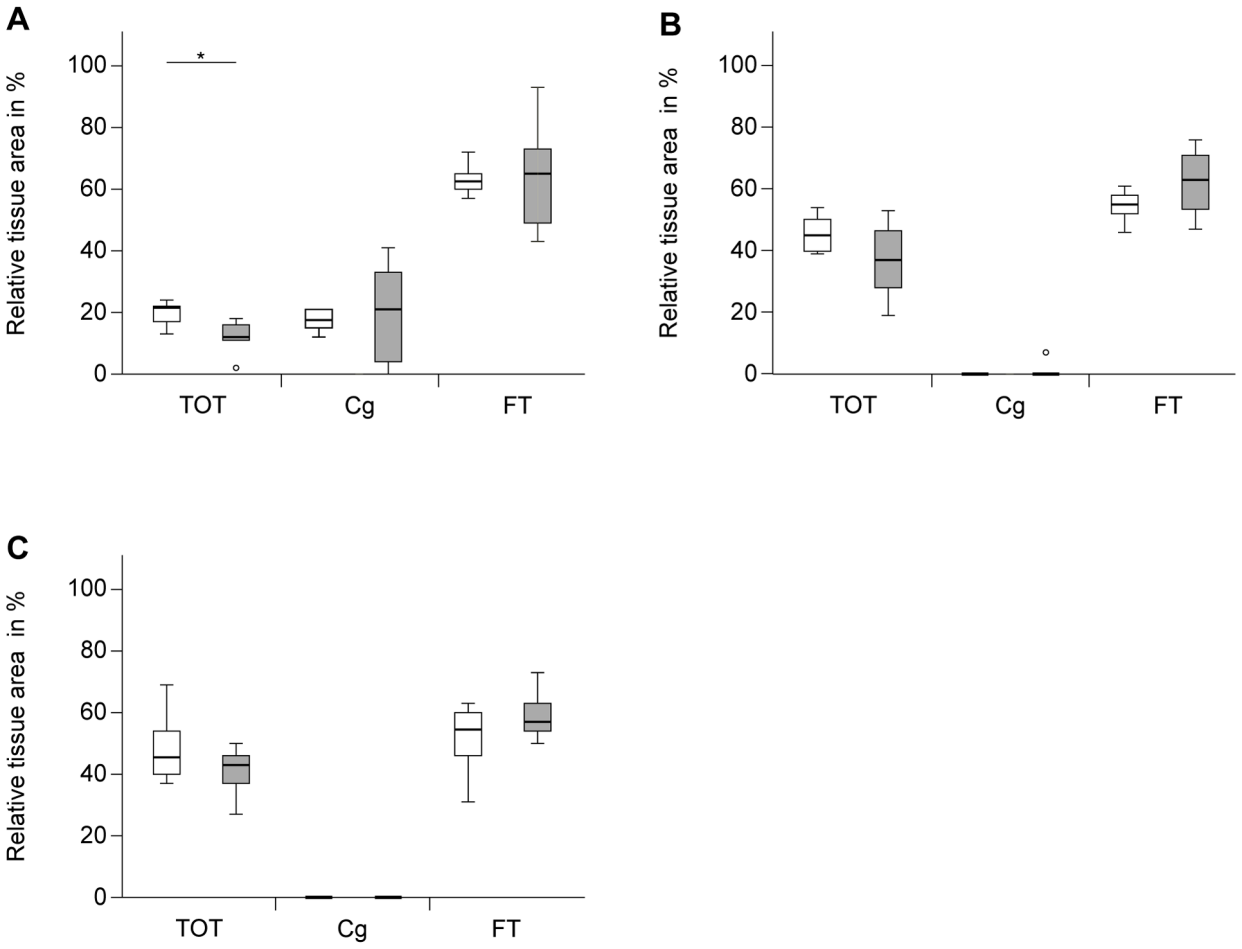
Histological evaluation of relative amounts of tissues in the fracture calli at different time points post fracture. WT: white columns, *Fzd9* ^−/−^: grey columns. All values are presented as median, interquartile ranges, minimum and maximum. n = 5–10; Mann-Whitney-U-test, *p<0.05. TOT: total osseous tissue, Cg: cartilage, FT: fibrous tissue. A: day 10, WT: n = 6, *Fzd9* ^−/−^: n = 5, B: day 24, WT: n = 7, *Fzd9* ^−/−^: n = 8, C: day 32, WT: n = 10, *Fzd*9^−/−^: n = 8.

**Figure 2:**
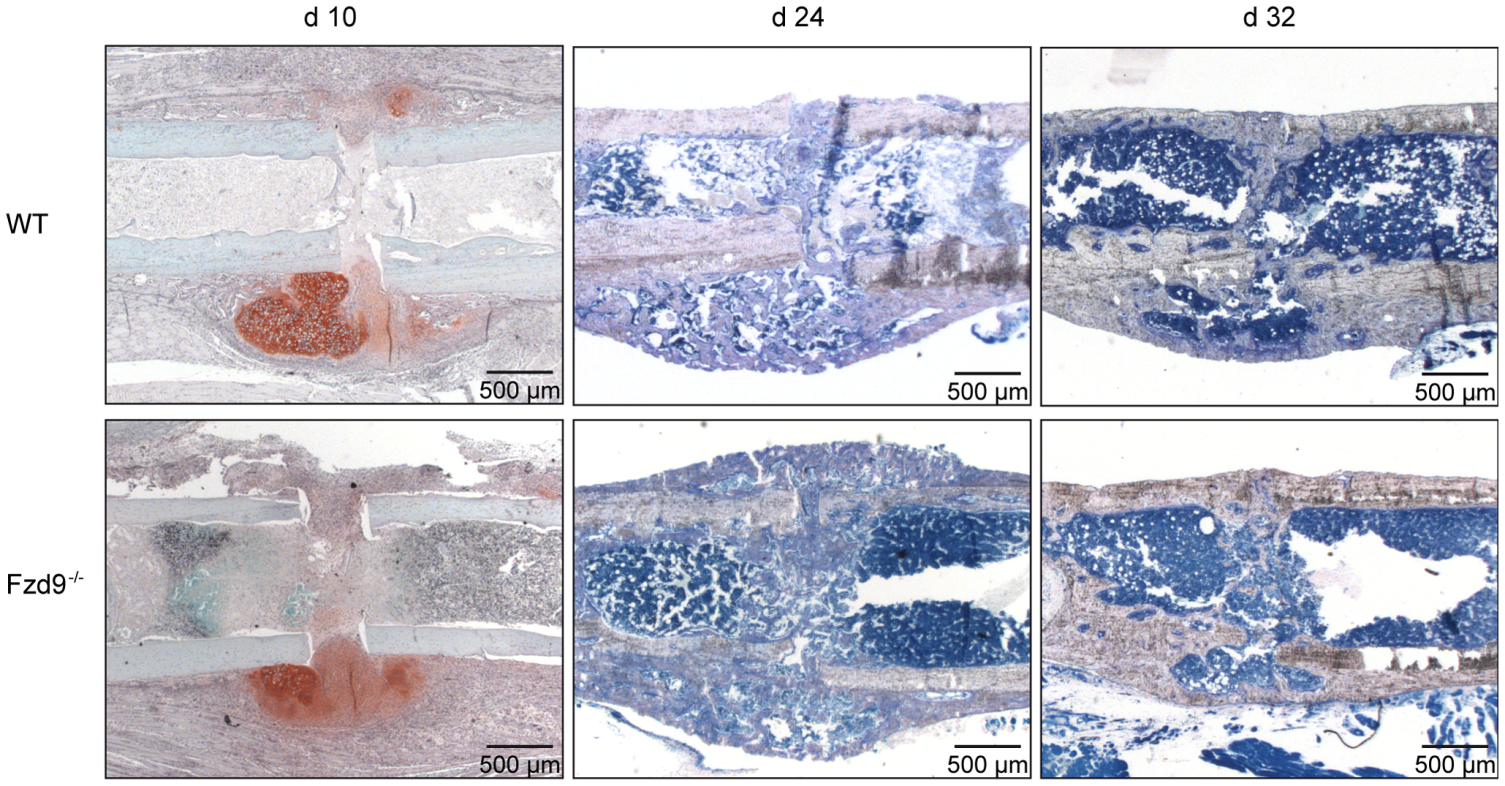
Representative histological sections of the fracture calli of WT and *Fzd9^−/−^* mice at different time points post fracture. The upper panel shows sections of osteotomized WT femurs and the lower one femurs of *Fzd9* ^−/−^. Decalcified sections 10 days post fracture were stained with Safranin O. Undecalcified femurs 24 and 32 days post fracture were stained with Giemsa.

**Figure 3:**
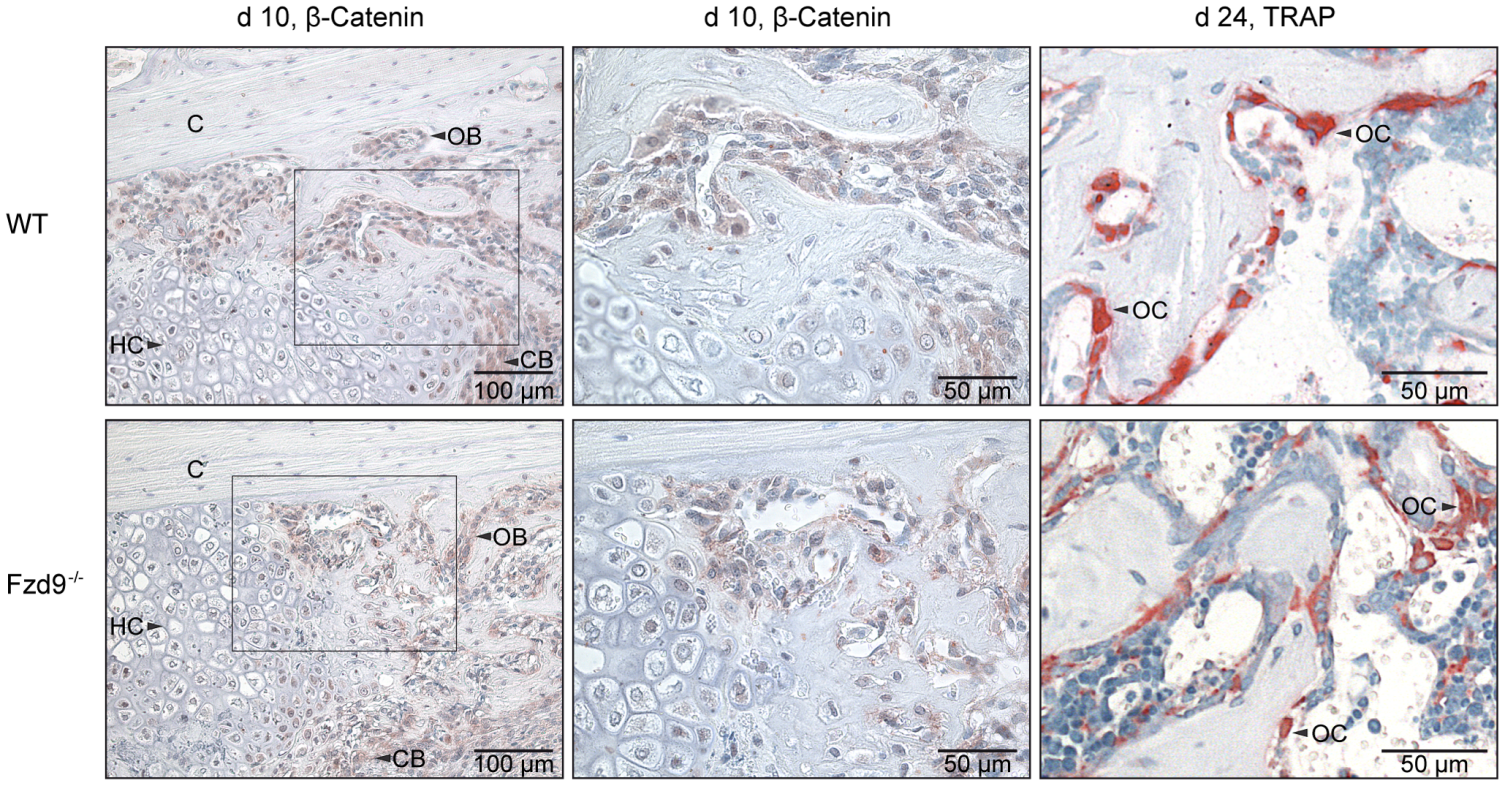
Immunostaining of β-catenin at day 10 and histochemical TRAP staining indicating osteoclasts at day 24 in the fracture calli. WT: upper panel, *Fdz9* ^−/−^: lower panel. β-catenin was expressed in osteoblasts (OB) and proliferating chondroblasts (CB) but to a lesser extend in hypertrophic chondrocytes (HC). C: cortex. There were no differences between both genotypes. Only TRAP positive cells with ≥2 nuclei were identified as osteoclasts (OC). TRAP-staining either revealed no significant differences between both genotypes.

**Figure 4:**
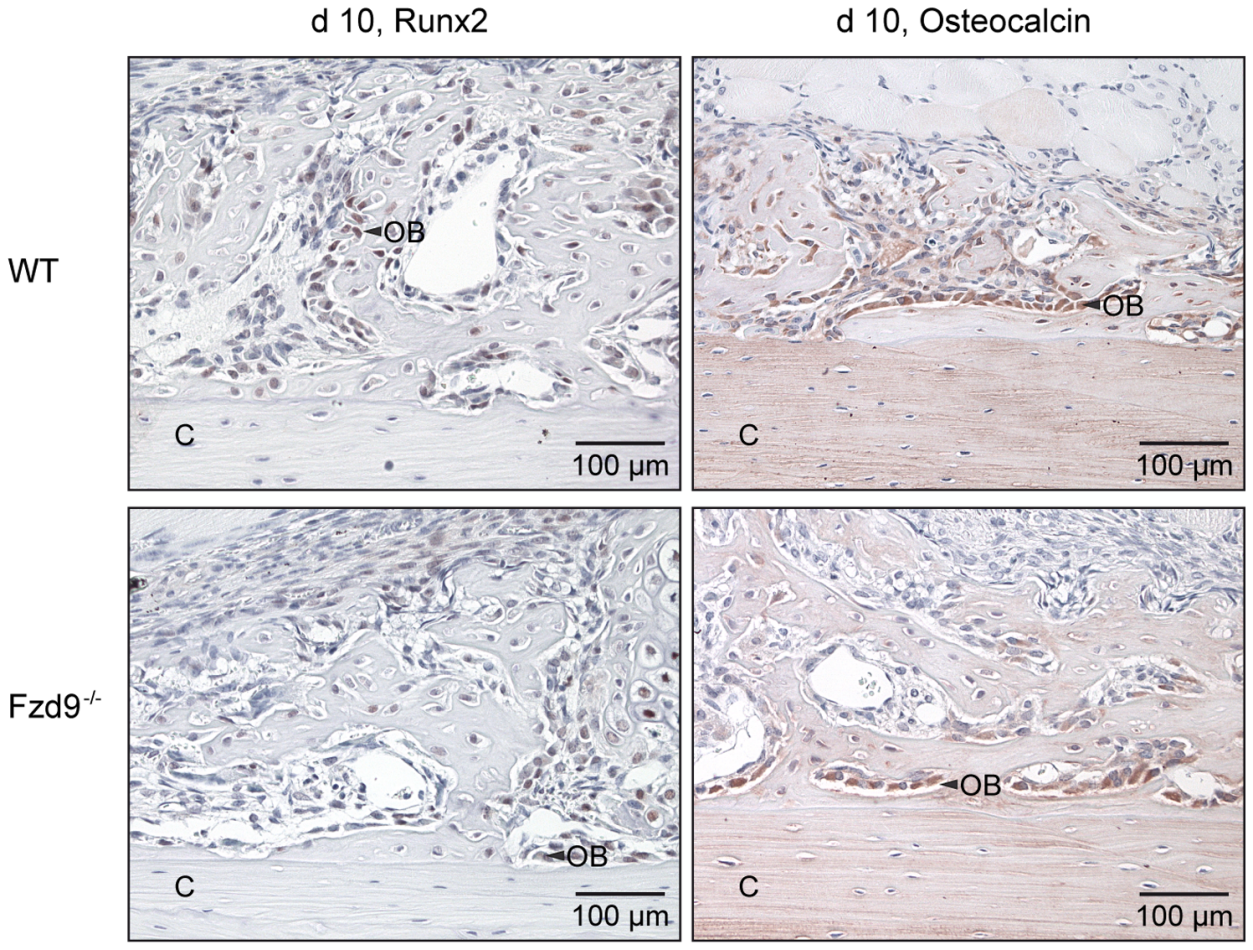
Immunostaining of osteoblast differentiation markers Runx2 and Osteocalcin at day 10. WT: upper panel, *Fdz9* ^−/−^: lower panel. Runx2 (*left*) was expressed mainly in the nucleus of preosteoblastic cells and osteoblasts (OB) located in the fracture callus. Osteocalcin (*right*) was mainly localized in the cytoplasma of osteoblasts near mineralized matrix and in the bone matrix. There were no differences in staining of Runx2 and Osteocalcin between both genotypes. C: cortex, OT: osteocytes.

**Figure 5:**
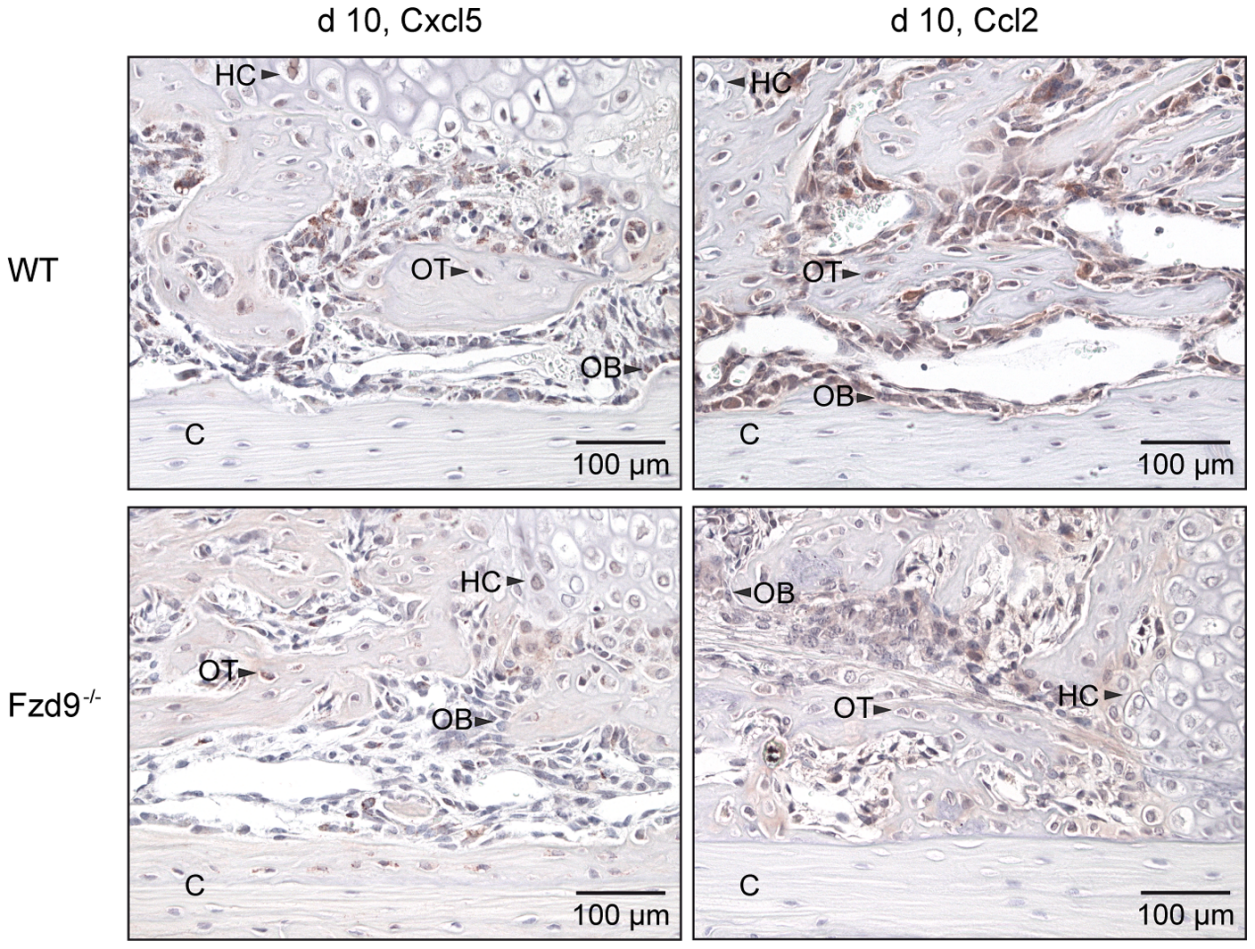
Immunostaining of chemokines Cxcl5 and Ccl2 at day 10. WT: upper panel, *Fdz9* ^−/−^: lower panel. C: cortex. Cxcl5 (*left*) and Ccl2 (*right*) were expressed by precursor cells, osteoblasts and chondrocytes in both genotypes, but staining was less intense in the absence of *Fzd9*. C: cortex, HC: hypertrophic chondrocytes, OB: osteoblasts, OT: osteocytes.

After 10 days, most of the callus was composed of cartilage and fibrous tissue. New bone formation, which started at some distance from the fracture gap near the periosteum, was significantly reduced by 45% (*p* = 0.043) in the absence of *Fzd9*, whereas the amount of cartilage was not significantly affected (Figures 1A and 2). The distribution and number of osteoclasts in the callus resorbing the mineralized cartilage during endochondral ossification and the woven bone during callus remodeling were not altered in the absence of *Fzd9* (1±0.7 and 1±0.2 OC/BS mm^−1^ in WT and *Fzd9* ^−/−^ mice, respectively). β-Catenin was found in the cytoplasm and the nucleus of osteoblasts as well as proliferating chondroblasts. Hypertrophic chondrocytes were barely stained. No differences in staining were detected between *Fzd9* ^−/−^ and WT mice (Figure 3). Runx2, an early osteoblast marker, was mainly found in the nucleus of preosteoblastic cells and osteoblasts located in the fracture callus (Figure 4). Osteocalcin, a late osteoblast differentiation marker, was mainly localized in the cytoplasma of osteoblasts near mineralized matrix and in the bone matrix (Figure 4). The expression of both differentiation markers was not affected by the absence of *Fzd9*. Because we previously observed a reduced expression of chemokines in *Fzd9^−/−^* osteoblasts [28], we immunostained Cxcl5 and Ccl2 in the fracture callus. Both chemokines were expressed by precursor cells, osteoblasts and chondroblasts in both genotypes, but staining seemed to be less intense in the absence of *Fzd9* (Figure 5).

**Figure 6:**
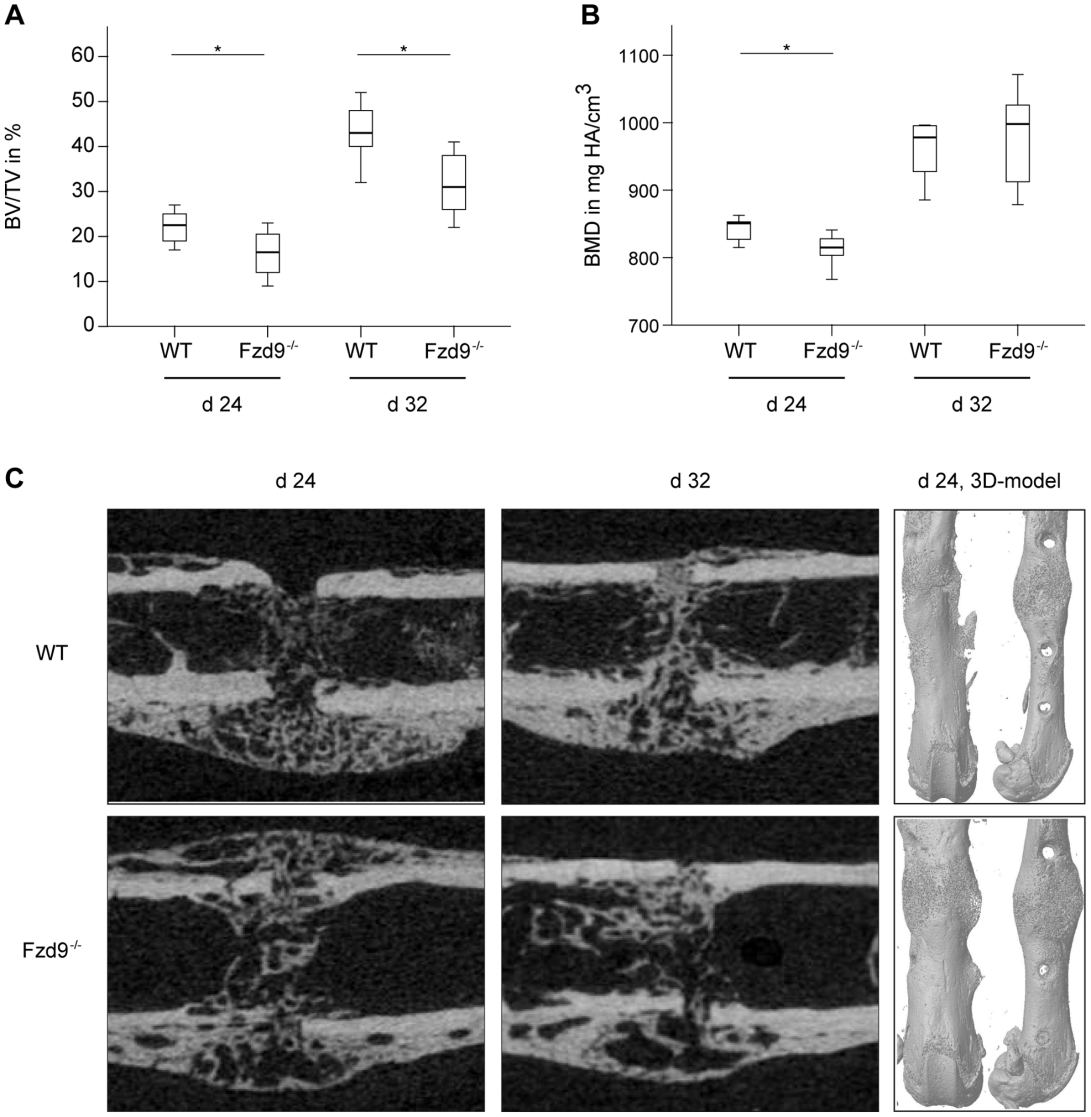
µCT evaluation of the fracture calli of WT and *Fzd9* ^−**/**−^ mice at days 24 and 32 post fracture. A: Bone volume/total volume (BV/TV). B: Bone mineral density (BMD) C: 3D-models of femurs at day 24 in anterior-posterior (left) and lateral view (right). The upper panel shows WT femurs and the lower panel *Fzd9* ^−/−^ femurs. *Fzd9* ^−/−^ mice exhibited a less mineralized callus at both investigated time points. At day 24, the geometry of the calli of *Fzd9* ^−/−^ mice was different from WT mice, resulting in an increased moment of inertia (*I_x_*) (see Figure 7C).

After 24 and 32 days, histology did not reveal any significant differences in tissue formation (Figures 1B, 1C and 2) or the number of osteoclasts (2±0.4 and 2±0.5 OC/BS mm^−1^ in WT and *Fzd9* ^−/−^ mice, respectively) (Figure 3), however, we did observe a trend towards a lower amount of newly formed bone in *Fzd9* ^−/−^ mice. At these late time points, the amount of cartilage was negligibly small in both genotypes, indicating that the cartilage to bone transformation was completed. µCT data confirmed the histology. BV/TV was significantly diminished in the fracture gap in the absence of *Fzd9* by 30% (*p* = 0.019) and by 21% (*p* = 0.004) after 24 and 32 days, respectively (Figure 6A). BMD was also significantly diminished in *Fzd9* ^−/−^ mice after 24 days (Figure 6B). The finding, that µCT analysis revealed significant differences between the genotypes at days 24 and 32, whereas histology did not, could be explained by the greater accuracy of three-dimensional µCT measurements in comparison to two-dimensional histology. Furthermore, in µCT-evaluation a global threshold was used to differentiate between mineralized and non-mineralized tissue, whereas in histomorphometry newly formed bone was identified by its characteristic morphology, thus also including less mineralized bone in the evaluation.

Taken together, our data suggest that in the absence of *Fzd9*, new bone formation but not cartilage formation in the fracture callus was significantly inhibited at all investigated time points.

### *Fzd9* Deficiency Impaired the Mechanical Competence of the Newly Formed Fracture Callus

**Figure 7:**
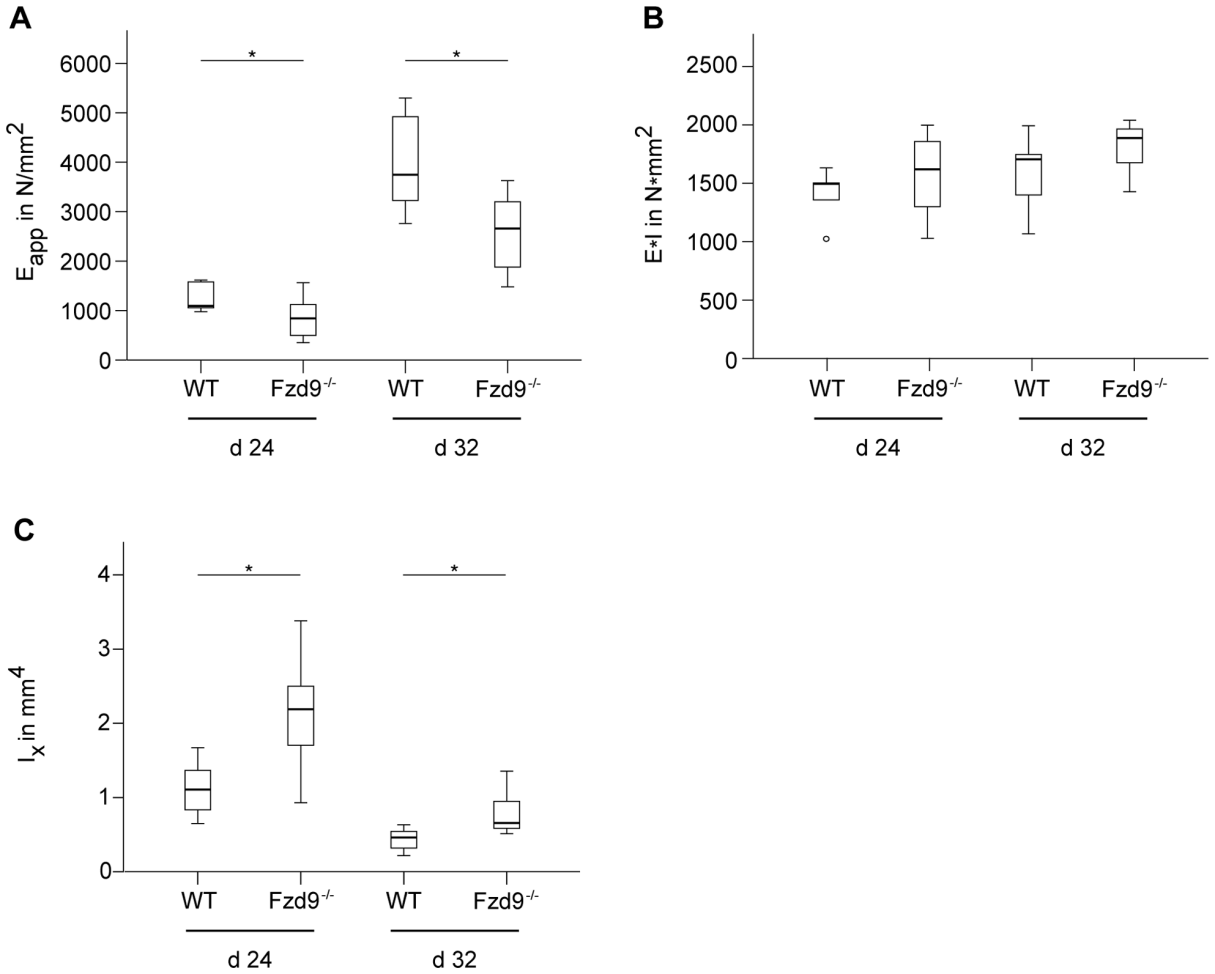
Mechanical characterization of the fracture calli of WT and *Fzd9* ^−**/**−^ mice at days 24 and 32 post fracture. All values are presented as median, interquartile ranges, minimum and maximum. n = 5–10; Mann-Whitney-U-test, *p<0.05. A: Apparent Young's Modulus *E*_app_ calculated as the ratio of flexural rigidity and moment of inertia, which describes the apparent material properties of the newly formed tissue. B: Flexural rigidity of the fracture calli measured by 3-point-bending test. C: Moment of inertia (*I_x_*) of the fracture calli measured by micro-computed tomography (µCT).

Confirming the histological and µCT data, the mechanical competence of the newly formed tissue in the fracture callus determined as the apparent Young's Modulus *E*_app_ was significantly decreased in the absence of *Fzd9* by 32% (*p* = 0.040) and 35% (*p* = 0.008) after 24 and 32 days, respectively (Figure 7A). *E*_app_, which is calculated as the ratio of flexural rigidity and moment of inertia, describes the apparent material properties of the inhomogeneous, newly formed callus tissue independently of the geometry of the fracture callus [32]. The decreased *E*_app_ in *Fzd9*-deficient mice resulted from the lower amount of mineralized bone in the callus (Figure 6). Notably, the flexural rigidity of the healed bones was not significantly different between both genotypes (Figure 7B). The flexural rigidity depends not only on the quality of the newly formed tissue (BV/TV, BMD) but also on the geometry of the callus, which is described by the moment of inertia. The moment of inertia in the bending axis was significantly increased in *Fzd9*-deficient mice (by 81%, *p* = 0.019, and by 77%, *p* = 0.004, after 24 and 32 days, respectively), indicating that the callus geometry was altered in these mice (Figures 6C and 7C). This results in a similar flexural rigidity for both genotypes, although the callus quality was significantly decreased in the absence of *Fzd9*.

## Discussion

Fzd9 belongs to the ten known transmembrane receptors of the Frizzled family, which participate in the canonical and non-canonical Wnt-signal transduction pathways [3], [5]. It is suggested that, in humans, FZD9 may be involved in the regulation of bone formation, because its deletion in the Williams-Beuren syndrome, a rare genetic disease with multiple manifestations, is associated with low bone mass [29], [30], [31]. Supporting the relevance of *Fzd9* in bone remodeling, it was recently demonstrated that the absence of *Fzd9* in mice resulted in osteopenia caused by an autonomous osteoblast defect. Because canonical Wnt/β-catenin signaling was not affected in *Fzd9*-deficient osteoblasts, it was suggested that *Fzd9* regulates bone mass via non-canonical Wnt-signaling pathways [28]. The data of the present study provide the first evidence that *Fzd9*, in addition to its role in physiological bone remodeling, regulates new bone formation in fracture healing. The fracture calli of *Fzd9*-deficient mice contained significantly less newly formed bone in the early and late healing periods, and exhibited inferior mechanical competence, implying that *Fzd9* positively regulates bone repair via β-catenin-independent Wnt-signaling pathways.

During the early phases of fracture healing, bone is predominantly formed by direct differentiation of committed osteoprogenitor cells and undifferentiated mesenchymal cells at some distance from the fracture gap, where the mechanical conditions and blood supply are adequate (intramembranous bone formation). Near the fracture, endochondral bone formation occurs by differentiation of mesenchymal cells into chondroblasts, which produce cartilaginous matrix. Later, the cartilage undergoes calcification and is transformed to bone when fracture healing progresses successfully [12], [13]. Our data demonstrated a significantly reduced amount of newly formed bone in both early and later fracture callus in the absence of *Fzd9*, indicating that direct intramembranous ossification and endochondral bone formation were both significantly compromised. This finding corresponds to the poor mechanical quality of the callus tissue at the later healing stages. It was previously demonstrated, that bone marrow stromal cells derived from *Fzd9*-knockout mice exhibited a diminished capacity to proliferate and form mineralized matrix, suggesting that an intrinsic osteoblast defect may be responsible for the osteopenia observed in these mice [28]. The disturbed osteoblast function also explains our present results of reduced bone formation in fracture healing. The molecular analysis of *Fzd9*-deficient osteoblasts showed that established differentiation markers, such as Runx2, Alpl and Bglap, were not affected [28]. This could be confirmed in the present study, as Runx2 and Osteocalcin were similarly expressed in the fracture callus of WT and *Fzd9* ^−/−^ mice. In contrast, *Fzd9* ^−/−^ primary osteoblasts displayed a reduced expression of several interferon-regulated genes [28], among them the ubiquitin-like modifier *Isg15*, which is involved in posttranslational modifications of multiple proteins [33]. It was postulated that *Isg15* might be one of the relevant downstream targets of *Fzd9* in the regulation of bone formation, because the impaired activity of the *Fzd9*-deficient osteoblast to form mineralized matrix could be rescued after transduction of an *Isg15* retrovirus and because *Isg15*-deficient mice displayed osteopenia [28]. However, the potential target proteins of *Isg15* in osteoblasts remain to be elucidated. Of great relevance for our present results may also be the fact that *Fzd9*-deficient primary osteoblasts displayed a lower expression of several chemokine-encoding genes, including *Ccl5*, *Ccl2* and *Cxcl5*, which were induced by the non-canonical Wnt agonist Wnt5a in WT osteoblasts, suggesting that they are direct targets of non-canonical Wnt signaling [28], [34]. In line with these data, the immunostaining of Cxcl5 and Ccl2 appeared to be reduced in the fracture callus of *Fzd9*-deficient mice. These chemokines not only play a crucial role in bone metabolism [35], [36], but also regulate fracture healing [37]. Recently, it was shown that inflammatory mediators, such as TNF-α, which are key regulators in the inflammatory phase of fracture healing [13], induce cytokine and chemokine production in mesenchymal stromal cells and osteoblasts via Wnt5a [34]. This underlines the critical role of non-canonical Wnt signaling in the regulation of inflammatory processes in bone. The reduced expression of chemokines by mesenchymal cells and osteoblasts may contribute to the pathomechanisms of delayed fracture healing in *Fzd9*-deficient mice, even though further studies are necessary to unravel the molecular effect of *Fzd9* in bone regeneration.

Using various mouse models with inducible ubiquitous or osteoblast-specific expression of null or stabilized β-catenin alleles, Chen et al. identified β-catenin as a key player in fracture healing [15]. Their results revealed that β-catenin positively regulated fracture healing when mesenchymal precursor cells had already been committed to the osteoblastic lineage. In contrast, β-catenin activation had to be precisely regulated in undifferentiated mesenchymal cells to allow successful bone repair, implicating a distinct role for canonical Wnt signaling in different healing phases [15]. Other studies confirmed the dominant role of canonical Wnt/β-catenin signaling, leading to the strategy of targeting this pathway to achieve improved bone repair [19], [20], [21], [22], [23]. Because our previous study revealed that *Fzd9* regulates osteoblast function via non-canonical pathways, whereas Wnt/β-catenin signaling was not affected [28], we were interested whether β-catenin was still present in the fracture callus of *Fzd9*-deficient mice. Actually, our results demonstrated that β-catenin was expressed in osteoblasts within the fracture callus in WT mice and that this expression level was not affected by the absence of *Fzd9*. This supports our previous findings [28] and provides the first evidence that, in addition to the canonical Wnt/β-catenin pathway, non-canonical Wnt signal transduction via *Fzd9* may be crucial for successful bone formation in fracture healing. The relevance of non-canonical Wnt pathways for bone repair is supported by other studies, demonstrating the up-regulation of non-canonical Wnt agonists, including Wnt4, Wnt5a or Wnt11, during healing [15], [18], [38].

Our results further showed that the amount of cartilage in the early fracture callus was unaffected by the absence of *Fzd9*, suggesting that non-canonical signaling via *Fzd9* may not be crucial for the differentiation of mesenchymal precursor cells towards the chondrogenic lineage. It is known that Wnt signaling can both positively and negatively regulate the different stages of chondrogenesis during skeletal development [4], [39], [40], however, the role of *Fzd9* has not been addressed. Additionally, the investigation of the regulation of cartilage formation in fracture healing by Wnt-signaling pathways is limited. To our knowledge, only one study specifically addressed this question by using Col2a1-ICAT mice, in which Wnt/β-catenin signaling is exclusively blocked in chondrocytes by preventing β-catenin and TCF/LEF forming the gene-transcription regulation complex [41]. The authors found delayed cartilage formation and chondrocyte hypertrophy in fracture healing of these mice, suggesting that β-catenin is essential for chondrogenesis and endochondral ossification in bone repair. This is supported by our own data, showing that β-catenin was, in addition to osteoblasts, particularly localized in proliferating chondrocytes, a finding confirmed by others [17]. These data indicate that Wnt/β-catenin signaling rather than non-canonical signaling may be important for cartilage formation in fracture healing.

We also found that even in the absence of *Fzd9* the cartilage in the callus was successfully degraded in the later phases of fracture healing, indicating undisturbed osteoclast activity. This was confirmed by our histological findings, demonstrating that in the absence of *Fzd9*, the number of osteoclasts in the fracture callus was not affected in the early or later healing stages. This is entirely in line with our previous findings of normal osteoclast number and activity in bone remodeling of *Fzd9*-deficient mice [28] and can be explained by the fact, that osteoclast activity is primarily regulated via the Wnt/β-catenin pathway, which controls the expression of *Tnfrsf11b* in osteoblasts, a gene encoding osteoprotegerin, an inhibitor of osteoclastogenesis [42], [43], [44].

In conclusion, our data provide the first evidence that *Fzd9*, in addition to its role in physiological bone remodeling, modulates new bone formation in fracture healing, indicating that bone-repair regulation is complex and involves both canonical and non-canonical Wnt-signaling pathways. The results of this study could have implications for the development of treatments of disturbed fracture healing, because Fzd proteins as serpentine receptors belong to the major class of target proteins for currently available drugs [27].

## Materials and Methods

All experiments were performed according to international regulations for the care and use of laboratory animals and were approved by the responsible ethical committee (No. 1026, Regierungspräsidium Tübingen, Germany). Twenty-one female *Fzd9*-deficient mice (University Medical Center Hamburg-Eppendorf, Germany) and 21 corresponding wildtype (WT) mice (129S6/SvEvTac, Taconic Farms Inc, New York, USA) were used [28]. Mice were housed in cages in groups of up to four animals and kept on a 14 h light and 10 h dark rhythm with water and food provided *ad libitum*. All mice received the same standard diet (R/M-H, V1535-300, Ssniff Spezialitäten GmbH, Soest, Germany).

### Surgery

The surgery was described previously in detail [45]. Briefly, at the age of 26 weeks all mice received a standardized osteotomy at the mid-shaft of the right femur under general anesthesia with 2% isoflurane (Forene, Abbott, Wiesbaden, Germany). The osteotomy was performed using a “Gigli” wire saw (diameter 0.4 mm, RISystem Inc., Davos, Switzerland) and stabilized with an external fixator (axial stiffness 3.0 N/mm, RISystem Inc., Davos, Switzerland). Mice received 25 mg/l tramalhydrochloride (Tramal, Gruenenthal GmbH, Aachen, Germany) as analgesic in the drinking water 1 day preoperatively until 3 days postoperatively. For antibiosis, a single dose of clindamycin-2-dihydrogenphosphate (45 mg/kg, Clindamycin, Ratiopharm, Ulm, Germany) was injected subcutaneously during surgery. After 10, 24 and 32 days, mice were sacrificed and the femurs carefully explanted (n = 5–10).

### Biomechanical Testing of the Fracture Callus

To determine the mechanical competence of the fracture callus as a functional parameter for fracture healing, the osteotomized femurs of mice euthanatized after 24 and 32 days post-surgery were subjected to a non-destructive three-point-bending test as described in detail previously [45]. Briefly, after fixator removal, the proximal end of the femur was fixed in an aluminum cylinder using a two-component adhesive (i-Cem^®^ Self-Adhesive, Heraeus Kulzer, Hanau, Germany). The cylinder was fixed in a materials testing machine (Z10, ZwickRoell, Ulm, Germany) with all degrees of freedom except rotation in the sagittal plane. The femoral condyles rested unfixed on a moveable distal bending support. An axial load of up to 4 N was applied on the cranio-lateral surface of the fracture callus at the femoral mid-shaft, creating a bending moment in the sagittal plane. The bending stiffness was calculated from the slope (*k*) of the linear region of the force-deflection curve. The distance between the load vector and the proximal (*a*) and distal (*b*) supports was considered when the calli were not located exactly in the middle of the supports (*L/2*). Flexural rigidity (E*I) was calculated according to E*I = k*(a^2^*b^2^/3L). Furthermore, the apparent Young's Modulus *E*_app_ of the fracture callus was calculated as the ratio of flexural rigidity and moment of inertia (*I_x_*) in the axis of bending (anterior-posterior direction) (*E*_app_ = E*I/*I_x_*), which was measured using micro-computed tomography (µCT).

### Micro-Computed Tomography (μCT)

After biomechanical testing, all femurs explanted 24 and 32 days post surgery were scanned using a μCT device (Skyscan 1172, Skyscan, Kontich, Belgium) at a resolution of 8 μm and a voltage of 50 kV and 200 μA. Two phantoms with defined density of hydroxylapatite (250 mg/cm^3^ und 750 mg/cm^3^) were scanned to convert the attenuation coefficients of the voxels into BMD. For analysis, the volume of interest (VOI) was defined as the region of the former osteotomy gap (CTAnalyser, Skyscan, Kontich, Belgium). In the total volume (TV), the average moment of inertia with respect to the bending axis (I_x_) was determined. A global threshold of 642 mg hydroxylapatite/cm^3^ [46] was used to distinguish between mineralized and non-mineralized tissue to determine the bone volume fraction (BV/TV) and the average BMD of the mineralized tissue.

### Histomorphometry

Twenty-four and 32 days post surgery the femurs were subjected to undecalcified histology by fixation in 4% formalin for at least 48 h and dehydration by increasing ethanol concentrations. Next, the femurs were embedded in methyl-methacrylate and 7-μm slices were cut and stained with Giemsa for tissue quantification. Decalcified histology of fractured femurs was carried out 10 days after surgery. Bones were fixed in 4% formalin for 2 days, decalcified in 20% EDTA (pH 7.2–7.4) for 8–14 days and embedded in paraffin after dehydration. Slices of 7 μm were obtained and stained with Safranin O.

The histological slices were examined using light microscopy (Leica DMI6000 B, Leica, Heerbrugg, Switzerland; Software MMAF Version 1.4.0 MetaMorph®, Leica) under 50-fold magnification. In the region of interest (ROI), relative amounts of total osseous tissue (TOT), cartilage (Cg) and fibrous tissue (FT) were determined. After 24 and 32 days the former osteotomy gap (endosteal and periosteal) was defined as the ROI according to μCT analysis, whereas after 10 days the region of the osteotomy and the periosteal callus between the inner pins of the fixator were considered as the ROI.

Osteoclasts were identified using histochemical staining for tartrate-resistant acid phosphatase (TRAP) at days 10 and 24 [47]. Cells with ≥3 nuclei residing on the bone surface and positive for TRAP staining were regarded as osteoclasts, and the number of osteoclasts per bone surface in the callus (OcN/BS) was evaluated. ROIs were defined according to the ROIs of the histological and μCT analyses after 10 and 24 days.

Immunostaining of β-catenin, Runx2, Osteocalcin, and chemokines Cxcl5 and Ccl2 were performed on paraffin-embedded sections from day 10 using a polyclonal anti-rabbit β-catenin antibody (EMD Millipore Corporation, Massachusetts, USA), anti-Runx2 antibody (Cell Signaling Technology Inc., Danvers, USA), anti-Osteocalcin antibody (Biorbyt Ltd., Cambridge, UK), anti-Cxcl5 antibody (Bioss Inc., Woburn, USA), and anti-Ccl2 antibody (Bioss Inc., Woburn, USA) respectively. After deparaffinization of sections in xylene and rehydration with methanol, nonspecific sites were blocked with 4% bovine serum albumin (BSA) and 0.1% Triton X-100 (Sigma-Aldrich, Steinheim, Germany) for 60 min at room temperature. Sections were incubated with primary antibody against β-catenin (1∶150 in 1% BSA and 0.1% Triton X-100), Runx2 (1∶50 in 1% BSA and 0.1% Triton X-100), Osteocalcin (1∶200 in 1% BSA and 0.1% Triton X-100), Cxcl5 (1∶100 in 1% BSA and 0.1% Triton X-100) or Ccl2 (1∶150 in 1% BSA and 0.1% Triton X-100) over night at 4°C. To detect the primary antibody, a secondary biotinylated goat-anti-rabbit antibody (Invitrogen, Life Technologies Corporation, Darmstadt, Germany) was added for 30 min at room temperature. After the addition of streptavidin for signal amplification over the period of 15 min (Zytomed Systems, Berlin, Germany), 3-amino-9-ethylcarbazol (Zytomed Systems, Berlin, Germany) was used as the chromogen. Finally, counterstaining with hematoxylin (Waldeck, Münster, Germany) was performed. The slices were analyzed under light microscopy at 200-fold magnification.

### Statistical Analysis

All values are given as the median, with interquartile ranges, minimum and maximum. Statistics software IBM SPSS Statistics 19.0 (SPSS Inc., Chicago, USA) was used. Data were compared using a non-parametric Mann-Whitney-U-test. The level indicating significance was *p*≤0.05.

## References

[1] Harada S, Rodan GA (2003) Control of osteoblast function and regulation of bone mass. Nature 423: 349–355. 12748654 10.1038/nature01660

[2] Rachner TD, Khosla S, Hofbauer LC (2011) Osteoporosis: now and the future. Lancet 377: 1276–1287. 21450337 10.1016/S0140-6736(10)62349-5 PMC3555696

[3] Baron R, Kneissel M (2013) WNT signaling in bone homeostasis and disease: from human mutations to treatments. Nat Med 19: 179–192. 23389618 10.1038/nm.3074

[4] Macsai CE, Foster BK, Xian CJ (2008) Roles of Wnt signalling in bone growth, remodelling, skeletal disorders and fracture repair. J Cell Physiol 215: 578–587. 18247365 10.1002/jcp.21342

[5] Kim JH, Liu X, Wang J, Chen X, Zhang H, et al (2013) Wnt signaling in bone formation and its therapeutic potential for bone diseases. Ther Adv Musculoskelet Dis 5: 13–31. 23514963 10.1177/1759720X12466608 PMC3582304

[6] Gong Y, Slee RB, Fukai N, Rawadi G, Roman-Roman S, et al (2001) LDL receptor-related protein 5 (LRP5) affects bone accrual and eye development. Cell 107: 513–523. 11719191 10.1016/s0092-8674(01)00571-2

[7] Little RD, Carulli JP, Del Mastro RG, Dupuis J, Osborne M, et al (2002) A mutation in the LDL receptor-related protein 5 gene results in the autosomal dominant high-bone-mass trait. Am J Hum Genet 70: 11–19. 11741193 10.1086/338450 PMC419982

[8] Boyden LM, Mao J, Belsky J, Mitzner L, Farhi A, et al (2002) High bone density due to a mutation in LDL-receptor-related protein 5. N Engl J Med 346: 1513–1521. 12015390 10.1056/NEJMoa013444

[9] Richards JB, Rivadeneira F, Inouye M, Pastinen TM, Soranzo N, et al (2008) Bone mineral density, osteoporosis, and osteoporotic fractures: a genome-wide association study. Lancet 371: 1505–1512. 18455228 10.1016/S0140-6736(08)60599-1 PMC2679414

[10] Semenov MV, He X (2006) LRP5 mutations linked to high bone mass diseases cause reduced LRP5 binding and inhibition by SOST. J Biol Chem 281: 38276–38284. 17052975 10.1074/jbc.M609509200

[11] Balemans W, Ebeling M, Patel N, Van Hul E, Olson P, et al (2001) Increased bone density in sclerosteosis is due to the deficiency of a novel secreted protein (SOST). Hum Mol Genet 10: 537–543. 11181578 10.1093/hmg/10.5.537

[12] Claes L, Recknagel S, Ignatius A (2012) Fracture healing under healthy and inflammatory conditions. Nat Rev Rheumatol 8: 133–143. 22293759 10.1038/nrrheum.2012.1

[13] Gerstenfeld LC, Cullinane DM, Barnes GL, Graves DT, Einhorn TA (2003) Fracture healing as a post-natal developmental process: molecular, spatial, and temporal aspects of its regulation. J Cell Biochem 88: 873–884. 12616527 10.1002/jcb.10435

[14] Caetano-Lopes J, Lopes A, Rodrigues A, Fernandes D, Perpetuo IP, et al (2011) Upregulation of inflammatory genes and downregulation of sclerostin gene expression are key elements in the early phase of fragility fracture healing. PLoS One 6: e16947. 21347301 10.1371/journal.pone.0016947 PMC3037947

[15] Chen Y, Whetstone HC, Lin AC, Nadesan P, Wei Q, et al (2007) Beta-catenin signaling plays a disparate role in different phases of fracture repair: implications for therapy to improve bone healing. PLoS Med 4: e249. 17676991 10.1371/journal.pmed.0040249 PMC1950214

[16] Dean DB, Watson JT, Jin W, Peters C, Enders JT, et al (2010) Distinct functionalities of bone morphogenetic protein antagonists during fracture healing in mice. J Anat 216: 625–630. 20298438 10.1111/j.1469-7580.2010.01214.x PMC2871998

[17] Zhong N, Gersch RP, Hadjiargyrou M (2006) Wnt signaling activation during bone regeneration and the role of Dishevelled in chondrocyte proliferation and differentiation. Bone 39: 5–16. 16459154 10.1016/j.bone.2005.12.008

[18] Hadjiargyrou M, Lombardo F, Zhao S, Ahrens W, Joo J, et al (2002) Transcriptional profiling of bone regeneration. Insight into the molecular complexity of wound repair. J Biol Chem 277: 30177–30182. 12055193 10.1074/jbc.M203171200

[19] Minear S, Leucht P, Jiang J, Liu B, Zeng A, et al (2010) Wnt proteins promote bone regeneration. Science translational medicine 2: 29ra30. 10.1126/scitranslmed.3000231 20427820

[20] Agholme F, Aspenberg P (2011) Wnt signaling and orthopedics, an overview. Acta Orthop 82: 125–130. 21438671 10.3109/17453674.2011.572252 PMC3235279

[21] Jawad MU, Fritton KE, Ma T, Ren PG, Goodman SB, et al (2013) Effects of sclerostin antibody on healing of a non-critical size femoral bone defect. J Orthop Res 31: 155–163. 22887736 10.1002/jor.22186

[22] Ominsky MS, Li C, Li X, Tan HL, Lee E, et al (2011) Inhibition of sclerostin by monoclonal antibody enhances bone healing and improves bone density and strength of nonfractured bones. J Bone Miner Res 26: 1012–1021. 21542004 10.1002/jbmr.307

[23] Agholme F, Li X, Isaksson H, Ke HZ, Aspenberg P (2010) Sclerostin antibody treatment enhances metaphyseal bone healing in rats. J Bone Miner Res 25: 2412–2418. 20499342 10.1002/jbmr.135

[24] Kim JB, Leucht P, Lam K, Luppen C, Ten Berge D, et al (2007) Bone regeneration is regulated by wnt signaling. J Bone Miner Res 22: 1913–1923. 17696762 10.1359/jbmr.070802

[25] Komatsu DE, Mary MN, Schroeder RJ, Robling AG, Turner CH, et al (2010) Modulation of Wnt signaling influences fracture repair. J Orthop Res 28: 928–936. 20063381 10.1002/jor.21078 PMC3412133

[26] Baron R, Hesse E (2012) Update on bone anabolics in osteoporosis treatment: rationale, current status, and perspectives. The Journal of clinical endocrinology and metabolism 97: 311–325. 22238383 10.1210/jc.2011-2332 PMC3275361

[27] Imming P, Sinning C, Meyer A (2006) Drugs, their targets and the nature and number of drug targets. Nature reviews Drug discovery 5: 821–834. 17016423 10.1038/nrd2132

[28] Albers J, Schulze J, Beil FT, Gebauer M, Baranowsky A, et al (2011) Control of bone formation by the serpentine receptor Frizzled-9. J Cell Biol 192: 1057–1072. 21402791 10.1083/jcb.201008012 PMC3063134

[29] Pober BR (2010) Williams-Beuren syndrome. The New England journal of medicine 362: 239–252. 20089974 10.1056/NEJMra0903074

[30] Schubert C (2009) The genomic basis of the Williams-Beuren syndrome. Cellular and molecular life sciences: CMLS 66: 1178–1197. 19039520 10.1007/s00018-008-8401-y PMC11131529

[31] Cherniske EM, Carpenter TO, Klaiman C, Young E, Bregman J, et al (2004) Multisystem study of 20 older adults with Williams syndrome. American journal of medical genetics Part A 131: 255–264. 15534874 10.1002/ajmg.a.30400

[32] Claes L, Blakytny R, Besse J, Bausewein C, Ignatius A, et al (2011) Late dynamization by reduced fixation stiffness enhances fracture healing in a rat femoral osteotomy model. J Orthop Trauma 25: 169–174. 21321508 10.1097/BOT.0b013e3181e3d994

[33] Zhao C, Denison C, Huibregtse JM, Gygi S, Krug RM (2005) Human ISG15 conjugation targets both IFN-induced and constitutively expressed proteins functioning in diverse cellular pathways. Proc Natl Acad Sci U S A 102: 10200–10205. 16009940 10.1073/pnas.0504754102 PMC1177427

[34] Rauner M, Stein N, Winzer M, Goettsch C, Zwerina J, et al (2012) WNT5A is induced by inflammatory mediators in bone marrow stromal cells and regulates cytokine and chemokine production. J Bone Miner Res 27: 575–585. 22162112 10.1002/jbmr.1488

[35] Wintges K, Beil FT, Albers J, Jeschke A, Schweizer M, et al (2013) Impaired bone formation and increased osteoclastogenesis in mice lacking chemokine (C-C motif) ligand 5 (Ccl5). J Bone Miner Res 28: 2070–2080. 23553711 10.1002/jbmr.1937

[36] Binder NB, Niederreiter B, Hoffmann O, Stange R, Pap T, et al (2009) Estrogen-dependent and C-C chemokine receptor-2-dependent pathways determine osteoclast behavior in osteoporosis. Nat Med 15: 417–424. 19330010 10.1038/nm.1945

[37] Alblowi J, Tian C, Siqueira MF, Kayal RA, McKenzie E, et al (2013) Chemokine expression is upregulated in chondrocytes in diabetic fracture healing. Bone 53: 294–300. 23262028 10.1016/j.bone.2012.12.006 PMC3767396

[38] Kakar S, Einhorn TA, Vora S, Miara LJ, Hon G, et al (2007) Enhanced chondrogenesis and Wnt signaling in PTH-treated fractures. J Bone Miner Res 22: 1903–1912. 17680724 10.1359/jbmr.070724

[39] Chun JS, Oh H, Yang S, Park M (2008) Wnt signaling in cartilage development and degeneration. BMB Rep 41: 485–494. 18682032 10.5483/bmbrep.2008.41.7.485

[40] Yates KE, Shortkroff S, Reish RG (2005) Wnt influence on chondrocyte differentiation and cartilage function. DNA Cell Biol 24: 446–457. 16008513 10.1089/dna.2005.24.446

[41] Huang Y, Zhang X, Du K, Yang F, Shi Y, et al (2011) Inhibition of beta-catenin signaling in chondrocytes induces delayed fracture healing in mice. J Orthop Res 30: 304–310. 21818768 10.1002/jor.21505 PMC3690117

[42] Glass DA 2nd, Bialek P, Ahn JD, Starbuck M, Patel MS, et al (2005) Canonical Wnt signaling in differentiated osteoblasts controls osteoclast differentiation. Dev Cell 8: 751–764. 15866165 10.1016/j.devcel.2005.02.017

[43] Holmen SL, Zylstra CR, Mukherjee A, Sigler RE, Faugere MC, et al (2005) Essential role of beta-catenin in postnatal bone acquisition. J Biol Chem 280: 21162–21168. 15802266 10.1074/jbc.M501900200

[44] Kramer I, Halleux C, Keller H, Pegurri M, Gooi JH, et al (2010) Osteocyte Wnt/beta-catenin signaling is required for normal bone homeostasis. Molecular and cellular biology 30: 3071–3085. 20404086 10.1128/MCB.01428-09 PMC2876685

[45] Röntgen V, Blakytny R, Matthys R, Landauer M, Wehner T, et al (2010) Fracture healing in mice under controlled rigid and flexible conditions using an adjustable external fixator. J Orthop Res 28: 1456–1462. 20872581 10.1002/jor.21148

[46] Morgan EF, Mason ZD, Chien KB, Pfeiffer AJ, Barnes GL, et al (2009) Micro-computed tomography assessment of fracture healing: relationships among callus structure, composition, and mechanical function. Bone 44: 335–344. 19013264 10.1016/j.bone.2008.10.039 PMC2669651

[47] Heilmann A, Schinke T, Bindl R, Wehner T, Rapp A, et al (2013) Systemic treatment with the sphingosine-1-phosphate analog FTY720 does not improve fracture healing in mice. J Orthop Res 31: 1845–1850. 23818033 10.1002/jor.22426

